# Effective Removal of Dyes from Wastewater by Osmanthus Fragrans Biomass Charcoal

**DOI:** 10.3390/molecules28176305

**Published:** 2023-08-29

**Authors:** Zhemin Xie, Sijie Diao, Ruizheng Xu, Guiyu Wei, Jianfeng Wen, Guanghui Hu, Tao Tang, Li Jiang, Xinyu Li, Ming Li, Haifu Huang

**Affiliations:** 1 Key Laboratory of Low-Dimensional Structural Physics and Application, Education Department of Guangxi Zhuang Autonomous Region, College of Science, Guilin University of Technology, Guilin 541004, China; 2 Guangxi Novel Battery Materials Research Center of Engineering Technology, Center on Nanoenergy Research, School of Physics Science and Technology, Guangxi University, Nanning 530004, China

**Keywords:** osmanthus fragrans biomass charcoal, adsorbent, high efficiency, dye adsorption, regeneration cycle

## Abstract

The exploration of low-cost, high-performance adsorbents is a popular research issue. In this work, a straightforward method that combined hydrothermal with tube firing was used to produce Osmanthus fragrans biomass charcoal (OBC) from low-cost osmanthus for dye adsorption in water. The study examined the parameters of starting concentration, pH, and duration, which impacted the process of adsorption of different dyes by OBC. The analysis showed that the adsorption capacities of OBC for six dyes: malachite green (MG, *C*_0_ = 800 mg/L, pH = 7), Congo red (CR, *C*_0_ = 1000 mg/L, pH = 8), rhodamine B (RhB, *C*_0_ = 500 mg/L, pH = 6), methyl orange (MO, *C*_0_ = 1000 mg/L, pH = 7), methylene blue (MB, *C*_0_ = 700 mg/L, pH = 8), and crystalline violet (CV, *C*_0_ = 500 mg/L, pH = 7) were 6501.09, 2870.30, 554.93, 6277.72, 626.50, and 3539.34 mg/g, respectively. The pseudo-second-order model and the Langmuir isotherm model were compatible with the experimental findings, which suggested the dominance of ion exchange and chemisorption. The materials were characterized by using XRD, SEM, FTIR, BET, and XPS, and the results showed that OBC had an outstanding specific surface area (2063 m^2^·g^–1^), with potential adsorption mechanisms that included electrostatic mechanisms, hydrogen bonding, and π-π adsorption. The fact that the adsorption capacity did not drastically decrease after five cycles of adsorption and desorption suggests that OBC has the potential to be a dye adsorbent.

## 1. Introduction

Organic dyes are widely utilized in the dyeing, textile, and food industries. Meanwhile, they provide bright colors to a wide range of items, but they also produce 600,000 tons of wastewater annually [1]. These dye effluents leak into the ground, which results in the contamination of aquatic creatures and harm to groundwater systems. Because of their excellent dyeing performance and straightforward synthesis procedure, azo dyes are frequently utilized in commercial dyestuffs. Many common azo dyes, such as malachite green (MG), Congo red (CR), methyl orange (MO), methyl blue (MB), and rhodamine B (RhB), are not readily decomposed, may be poisonous, and may be hazardous to human health [2]. Methods of treating dyes include electrocatalytic degradation, microbial degradation, adsorption, chemical oxidation, and coagulation or flocculation [3,4,5,6,7]. Many approaches are unavailable for large-scale use due to financial and environmental limitations. Among them, the adsorption method [8] is widely used because of its ease of use, affordability, and lack of secondary contamination.

Many materials can be used as adsorbents, including bentonite [9], metal–organic frameworks [10], red mud [11], zeolite [12], chitosan [13], cellulose [14], fly ash [15], and porous carbon [16]. Carbon compounds, such as carbon quantum dots [17], graphene [18], activated carbon [19], and biomass carbon [20], have large numbers of functional groups and vacancies and large specific surface areas. Carbon derived from biomass is the most notable of them. Because biomass carbon is inexpensive, has strong charge mobility [21], and has surface tunability [22], it has taken on many different forms. A WS-CA-AM adsorbent was prepared by Liu et al. [23] by using functionalized straw, and it had adsorption capacities of 3053.48 and 120.84 mg·g^−1^ for methyl orange (MO) and methylene blue (MB), respectively. Using lychee peel, Wu et al. [24] created an HLP adsorbent material that had adsorption capacities of 404.4 and 2468 mg·g^−1^ for Congo red and malachite green, respectively. Jiang et al. [25] adsorbed MB with 98.56% efficiency by using carbon generated from algal biomass. The preparation conditions of different materials have an impact on their adsorption performance. Research on cost-effective, high-performance biochar is still very much in demand [26]. According to incomplete statistics, Guilin is home to around 200,000 osmanthus plants. The flowering osmanthus plant is used for food, medicine, and other purposes, in addition to air purification [27]. However, significant portions of the osmanthus blooms fall off each year during blossoming, making it impossible to utilize them twice and polluting the environment. After deriving carbon compounds from osmanthus, Quan et al. [28] discovered that they had an amorphous, many-pore structure that could be used in electrochemistry. By optimizing the pore structure, the porous carbon (OC) material generated from osmanthus, which was developed by Zou et al. [29], had a three-dimensional interpenetrating framework and could be employed as an electrode material. The selection of adsorbents was mostly based on factors such as ease of manufacture and inexpensive cost, with superior pore structure being crucial for dye adsorption.

For the purpose of the adsorption of non-degradable azo dyes, such as malachite green (MG), congo red (CR), methyl orange (MO), methylene blue (MB), rhodamine B (RhB), and crystalline violet (CV), in aqueous humor, Osmanthus fragrans biomass charcoal (OBC) was prepared in this work by using a combination of hydrothermal and tube-firing methods. This method is low-cost and highly effective while preserving resources and protecting the environment from pollution. We examined the effects of starting concentration, time, and pH on the adsorption process in order to gain a better understanding of the adsorption mechanism. Following that, an adsorption isotherm model and an adsorption kinetic model were fitted to the experimental data, along with a number of biochar characterizations from both before and after the adsorption of dyes. Additionally, the regeneration cycle performance of osmanthus biochar was investigated.

## 2. Results and Discussion

### 2.1. Material Characterization

**Figure 1:**
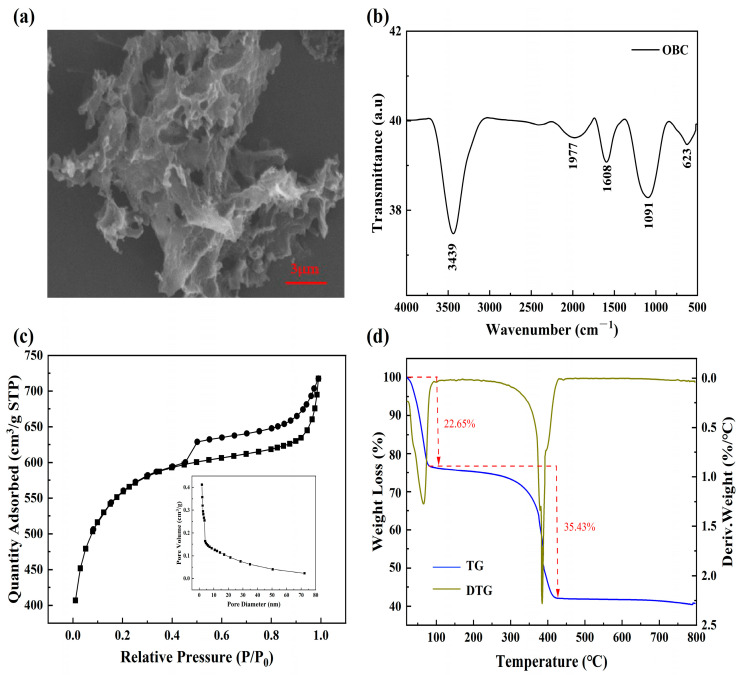
(**a**) SEM images of the OBC, (**b**) FTIR spectra, (**c**) pore size distribution (illustration) and nitrogen adsorption/desorption isotherms of the OBC, and (**d**) TG-DTG curves of the OBC.

As shown in Figure 1a, an SEM image of the morphology of OBC revealed that its surface was uneven and rough, creating a porous structure with varying pore diameters. This was because the carbon material was calcined at high temperatures, which produced CO_2_, and the alkali activation stage reaction released CO gas, which resulted in numerous irregular pore structures and offered more active adsorption sites for the adsorption of dyes. In Figure 1b, the surface functional groups of OBC determined with FTIR are displayed. The stretching vibration of -OH was represented by a significant absorption peak of about 3439 cm^−1^ [30]. Absorption peaks also appeared around 1977, 1608, and 1091 cm^−1^, suggesting that the OBC was rich in C=O, C=C, and C-O groups. The absorption peak at 623 cm^−1^ was attributed to the bending vibration of -CH [31], and these groups played an important role in the subsequent adsorption of dyes.

In Figure 1c, the adsorption–desorption isotherms of the OBC reflect the area of the surface and the pore characteristics. The average pore size of BET was 2.15 nm, and the maximum BET-specific surface area was 2063 m^2^·g^−1^. It displayed a mesopore lag ring pattern of H4 and type IV isotherms, suggesting that both mesopores and micropores made up the OBC. The gas adsorption quickly increased when P/P_0_ < 0.4, most likely as a result of the high content of microporous structures in the OBC. Nitrogen gradually filled the entire microporous position and moved into the mesoporous position when P/P_0_ increased. When P/P_0_ > 0.9, the gas adsorption amount still increased, which indicated that there were still macroporous structures in the OBC. It was clear that OBC had a highly irregular pore structure since the adsorption–desorption isotherms did not overlap to create a hysteresis loop and no obvious saturation states were seen. Thus, the OBC had a large number of irregular pore structures in addition to many mesopores, micropores, and a few macropores. The TGA curve of the OBC under a nitrogen atmosphere is shown in Figure 1d, which demonstrates the stages of the material’s weight loss as the temperature rose. There were two phases to the mass decrease. The mass loss of the OBC was 22.6% during the first stage, which was from ambient temperature to around 70 °C. This was because when the OBC powder was placed in a room-temperature environment, it absorbed the water molecules into the airspace, and its free and bound water evaporated rapidly [32]. The weight loss curve was dramatically altered after the second pyrolysis, which happened at 380 °C. This meant that the organic component in the OBC began to oxidatively break down, and the mass of the OBC considerably dropped. After this, the mass of the OBC equilibrated, indicating that the thermal decomposition was essentially complete.

### 2.2. Batch Adsorption Experiments

#### 2.2.1. Effect of Reaction Time and Adsorption Kinetics

The adsorption kinetic curves show the relationship between the equilibrium time and the adsorption capacity of the OBC for the adsorbed dyes. The following steps were taken: addition of 5 mg of OBC to conical flasks containing 50 mL of dye (MG, CR, MB, MO, RhB, and CV) and shaking on a thermostatic shaker (303 K, 180 rpm). This was followed by filtration through a 13 mm × 0.45 μm PTFE filter and measurement of the residual concentration of the dyes by using UV. To examine the connection between the adsorption time and equilibrium adsorption quantity, the experimental data were fitted using a pseudo-first-order adsorption kinetic model (Equation (1)) and a pseudo-second-order adsorption kinetic model (Equation (2)).

(1) $$\log\left(Q_{e}-Q_{t}\right)=\log Q_{e}-\frac{K_{1}}{2.303}t$$

(2) $$\frac{t}{Q_{t}}=\frac{1}{k_{2}Q_{e}^{2}}+\frac{t}{Q_{e}}$$

where the terms *Q_t_* (mg·g^−1^) and *Q_e_* (mg·g^−1^), respectively, stand for the adsorption capacity at time *t* and the adsorption equilibrium capacity. The adsorption time is *t* (min). The rate constants are denoted by the symbols *k*_1_ (min^−1^) and *k*_2_ (g·mg^−1^·min^−1^).

**Figure 2:**
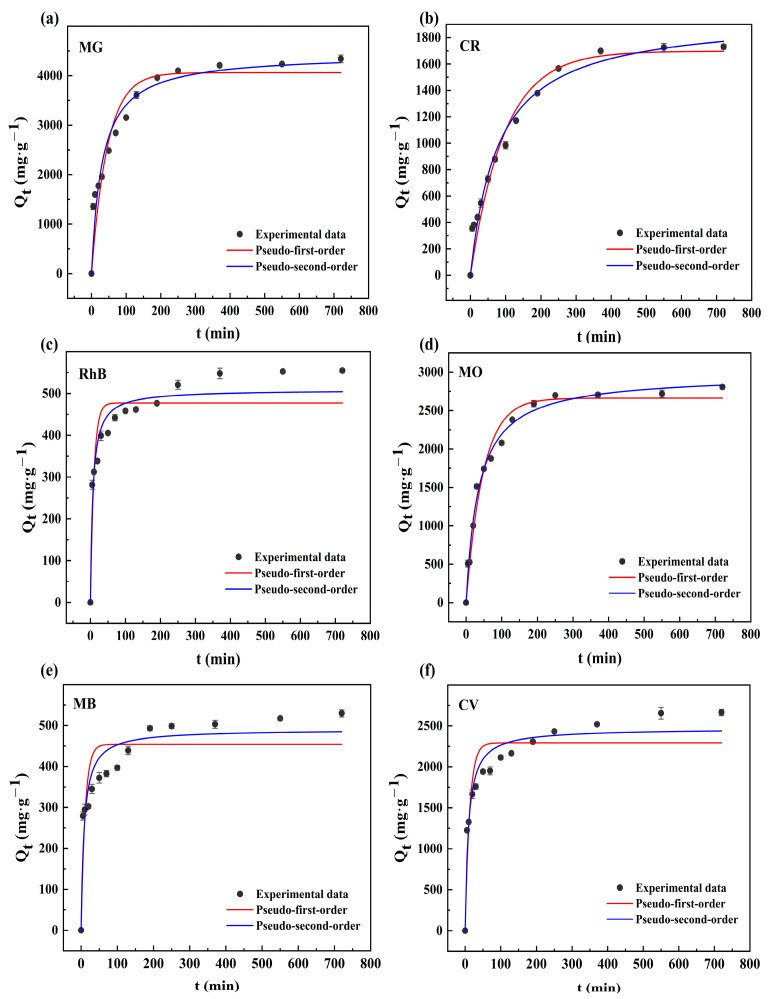
Pseudo-first-order and pseudo-second-order equation fitting for the OBC’s adsorption of the MG (**a**), CR (**b**), RhB (**c**), MO (**d**), MB (**e**), and CV (**f**) dyes.

As shown in Figure 2, the OBC adsorbed various dyes MG (Figure 2a), CR (Figure 2b), RhB (Figure 2c), MO (Figure 2d), MB (Figure 2e), and CV (Figure 2f), and different time intervals were utilized to collect the supernatant (5, 10, 20, 30, 50, 70, 100, 130, 190, 250, 370, 550, and 720 min) and to measure the concentration with a UV spectrophotometer. Due to the many pore structures of the OBC’s surface, the dye molecules were given a significant number of active adsorption sites when they initially came into contact with it, which caused the adsorption capacity to quickly grow. Over time, this led to a steady rise in adsorption capacity, and the majority of the active adsorption sites were gradually occupied by dye molecules. When the active adsorption sites of the OBC reached saturation, the curve tended to equilibrium, and it took around 300 min for the six dyes to reach adsorption equilibrium. The three dyes MG, CR, and MO had well-fitting rate equations, and the growth of the adsorption capacity at corresponding sampling times was relatively uniform. The fitted curves for the remaining RhB, MB, and CV were marginally below the final adsorption capacity, which was caused by the rapid increase in the adsorption capacity at the second sampling point. When the OBC was added to the dye solution, a diffusion process occurred. The thermostatic oscillator’s vibration sped up this diffusion by increasing the area in contact with the dye molecules, causing them to occupy the majority of the active adsorption sites, and rapidly increasing the adsorption capacity [25]. Furthermore, within the first 100 min, these dyes were heavily sampled, which also affected the curve fitting that came after [33]. With the exact parameters listed in Table 1, the experimental data were fitted to the pseudo-first-order and pseudo-second-order kinetic models. The pseudo-first-order kinetic model correlation coefficient (*R*^2^) was obviously lower than the pseudo-second-order kinetic model when comparing the two models. As per the suggested pseudo-second-order kinetics, there was a positive correlation between the concentration and the reaction rate. In addition, the adsorption capacities of MG, CR, RhB, MO, MB, and CV when fitted by the pseudo-second-order kinetic model were 4443.008, 1966.689, 508.968, 2969.427, 490.371, and 2468.242 mg/g, respectively, and they were closer to the experimental data (6501.09, 2870.30, 554.93, 6277.72, 626.50, and 3539.34 mg/g), which showed that the experimental data were more consistent with the pseudo-second-order kinetic model, suggesting that the whole adsorption process was dominated by ion exchange or chemisorption [34].

#### 2.2.2. Effects of Initial Concentration and Isotherms

The adsorption isotherms showed the relationship between the adsorption of different concentrations of dyes by the OBC and the adsorption capacities. Three isotherm models those of Langmuir (Equation (3)), Freundlich (Equation (4)), and Temkin (Equation (5)) were used to fit the experimental data and investigate the relationship between concentration and adsorption capacity when adsorption reached equilibrium. By using the Langmuir model, which provided a good match with the experimental data, the separation factor (*R_L_*) was calculated with Equation (6).

(3) $$\frac{C_{e}}{Q_{e}}=\frac{C_{e}}{Q_{m}}+\frac{1}{Q_{m}k_{L}}$$

(4) $$\ln Q_{e}=\ln k_{F}+\frac{1}{n}\ln C_{e}$$

(5) $$Q_{e}=B\ln K_{T}+B\ln C_{e}$$

(6) $$R_{L}=\frac{1}{1+K_{L}\cdot C_{0}}$$

where *Q_e_* (mg·L^−1^) represents the adsorption capacity at adsorption equilibrium, *C_e_* (mg·L^−1^) stands for the equilibrium concentration of the dye, and *Q_m_* (mg·L^−1^) stands for the maximum adsorption capacity in the Langmuir model. *K_L_* (L·mg^−1^) and *K_F_* ((mg·g^−1^)·(L·mg^−1^)^1/*n*^) are the Langmuir and Freundlich adsorption equilibrium constants, respectively, where *n* is the dimensionless constant for the Freundlich model. *K_T_* (L·g^−1^) and B (J·mol^−1^) indicate the Temkin equilibrium binding constant and isotherm constant, respectively. The gas constants *R* and *T* are the temperatures in Kelvin and *K*, respectively. The value of *R_L_* denotes the affinity of the adsorbent for the dyes. With *R_L_* > 1 indicating poor adsorption, 0 < *R_L_* < 1 indicating good adsorption, *R_L_* = 0 indicating irreversible adsorption, and *R_L_* = 1 indicating linear adsorption.

**Figure 3:**
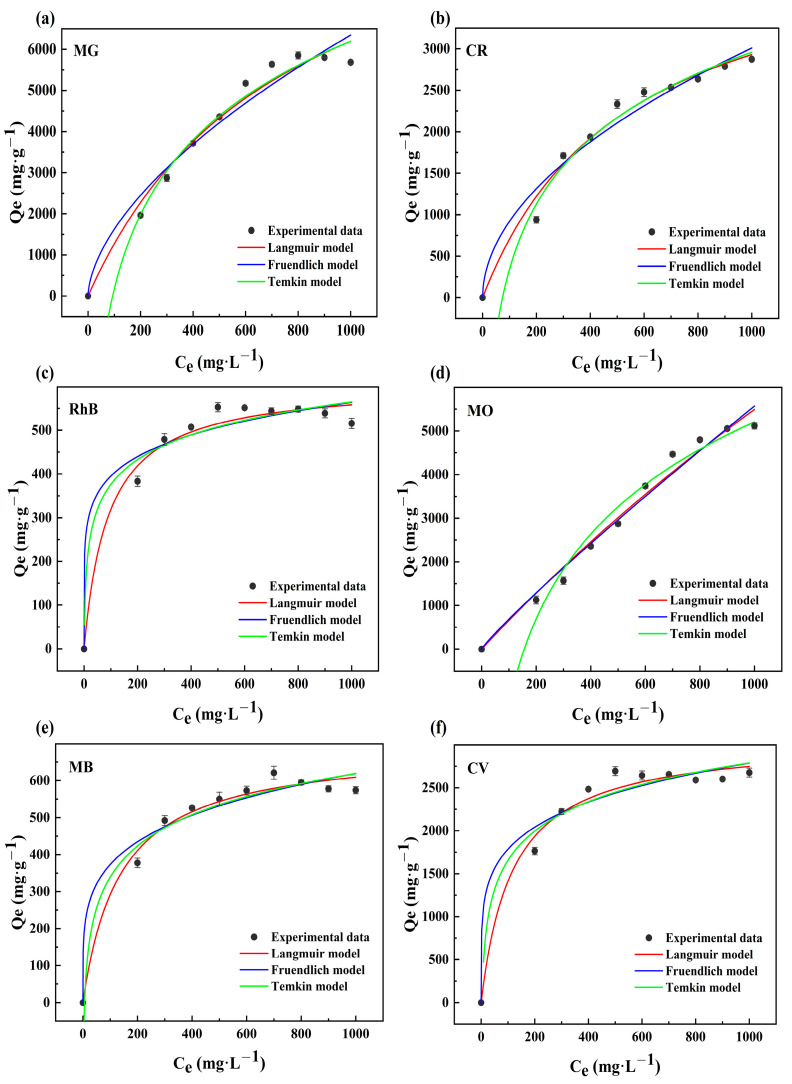
Fitting of the Langmuir, Freundlich, and Temkin curves for the OBC’s adsorption of MG (**a**), CR (**b**), RhB (**c**), MO (**d**), MB (**e**), and CV (**f**).

The adsorption capabilities of the OBC for the six dyes MG (Figure 3a), CR (Figure 3b), RhB (Figure 3c), MO (Figure 3d), MB (Figure 3e), and CV (Figure 3f) at various concentrations (200, 300, 400, 500, 600, 700, 800, 900, and 1000 mg·L^−1^) are presented in Figure 3. The adsorption capacity increased as the concentration of the dye rose, but as long as the active adsorption sites were occupied, the adsorption capacity tended to equilibrium and even seemed to be somewhat decreased. This was due to the fact that the dye molecules that were adsorbed on the OBC surface occupied the surface layer’s pore structure. However, when the thermostatic oscillator kept oscillating, some of the insecure dye molecules became dislodged, which reduced the OBC’s adsorption capacity. The figure also demonstrates that the optimal adsorption concentration for each dye was different, and the optimal adsorption concentrations for MG, CR, RhB, MO, MB, and CV were 800, 1000, 500, 1000, 700, and 500 mg·L^−1^, respectively. This was due to the fact that the density of each dye molecule was not the same, and the number of entries into the OBC pores would not be the same. This, combined with the fact that every dye had a particular environment, led to the varying adsorption capabilities at the various concentrations. The specific inputs for fitting the Langmuir, Freundlich, and Temkin models are listed in Table 2. When the *R*^2^ values of the three models were compared, the *R*^2^ value of the Langmuir model was the closest to 1, which indicated that the OBC’s adsorption of various dyes was more consistent with the Langmuir model. This indicated that on a homogeneous surface with specific adsorption sites in the OBC, dye monolayer adsorption took place [35]. The dimensionless separation factor (*R_L_*), which was estimated and used to determine if the adsorption process was thermodynamically favorable or not, was in the range of 0–1, suggesting that the OBC was beneficial for the adsorption of the various dyes. Additionally, every value of 1/n was less than one, meaning that every dye was effectively adsorbed. According to the Temkin model, *R*^2^ > 0.9, indicating that electrostatic interactions may have been engaged in the entire adsorption process [36].

#### 2.2.3. Effect of Different pH

**Figure 4:**
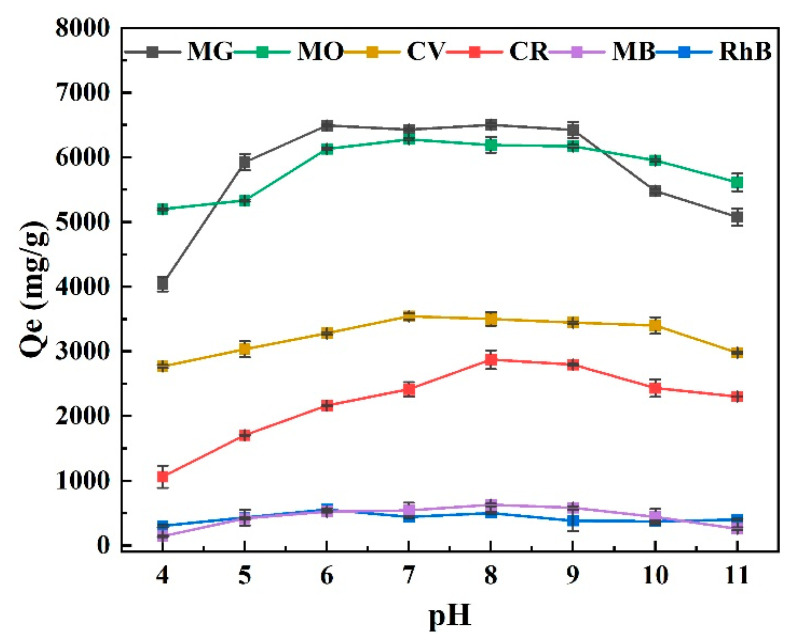
Effects of pH on OBC’s adsorption capacity for MG, CR, RhB, MO, MB, and CV dyes.

The degree to which OBC absorbed dyes depended critically on its pH level. The ability of OBC to bind six dyes (MG, CR, RhB, MO, MB, and CV) at different pH levels was compared to the dye adsorption, as shown in Figure 4. The high concentration of H^+^ in the solution with too low a pH caused the dye solutions’ adsorption capabilities to be poor at low pH values. When OBC was added to the dye, it first came into contact with H^+^, which competed with the dye molecules for the active adsorption sites, resulting in a low adsorption capacity. The deprotonation of the OBC’s reactive groups, which raised the negative charge density and encouraged more active interaction with the dye molecules, enhanced the adsorption capacity as the pH rose [37]. The adsorption capacity for RhB reached a maximum at a pH value of 6, which may have been because the dye was an anionic one and electrostatically adsorbed H^+^ on the OBC’s surface [38]. The adsorption capacities of MO, CV, and MG reached a maximum at pH 7, and the adsorption capacities of CR and MB reached a maximum at pH 8. At pH 9–11, all of the OBC adsorption capabilities for dye adsorption dropped drastically, indicating that the adsorption capacity of OBC for dye molecules may have resulted from the immobilization of certain dye molecules inside the OBC’s structure in addition to the interactions between functional groups [39]. The pore structure of the OBC may have been harmed by the very alkaline environment, which damaged the surface active sites and reduced the adsorption capabilities.

### 2.3. Adsorption Mechanism

**Figure 5:**
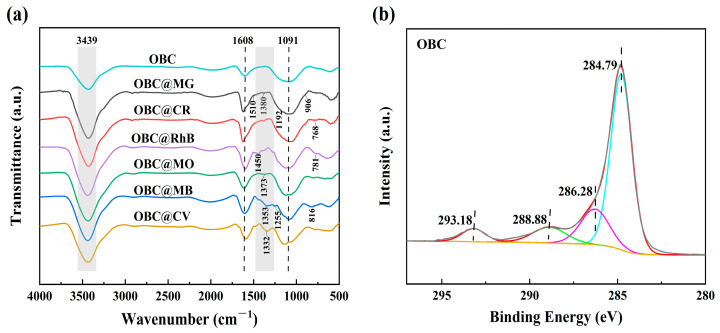
(**a**) FTIR spectra after adsorption (MG, CR, RhB, MO, MB, and CV). (**b**) High-resolution C 1s XPS spectra of OBC.

The FTIR patterns of OBC following the adsorption of the six dyes MG, CR, RhB, MO, MB, and CV are presented in Figure 5a. The appearance of some new characteristic peaks showed that the dye molecules were successfully adsorbed on the OBC (MG (1510, 1380, 1192, and 906 cm^−1^), CR (1115 and 768 cm^−1^), RhB (1450 and 761 cm^−1^), MO (1373 cm^−1^), MB (1353, 1255, and 816 cm^−1^), and CV (1332 cm^−1^)). The peak centered at 3439 cm^−1^ shifted as the dye molecules adsorbed. The -OH group was able to adsorb the dyes through a variety of mechanisms, such as the formation of hydrogen bonds [40], which could result in physical adsorption, or by functioning as a Lewis base and donating electron pairs to nearby dye molecules, which could result in the formation of chemical bonding between the dyes and the OBC, leading to adsorption. Furthermore, the density of negative charges on the surface of the OBC was increased by the presence of -OH groups, and positively charged dye molecules were drawn to the dense concentration of negative charges [41]. All of the absorption peaks at 1608 cm^−1^ were displaced [42], suggesting that bond interactions may have been involved in dye adsorption. This was because the dye molecule combined with the OBC was aromatic, and bond stacking occurred between the dye molecule’s aromatic ring structure and the benzene ring. In Figure 5b, the high-resolution C 1s spectra of the OBC are shown. These could be broken down into peaks at 284.79 eV with the cyan curve (C-C/C=C), 286.28 eV with the magenta curve (C-O), 288.88 eV with the green curve (O-C=O), and 293.18 eV with the blue curve (π-π).

**Figure 6:**
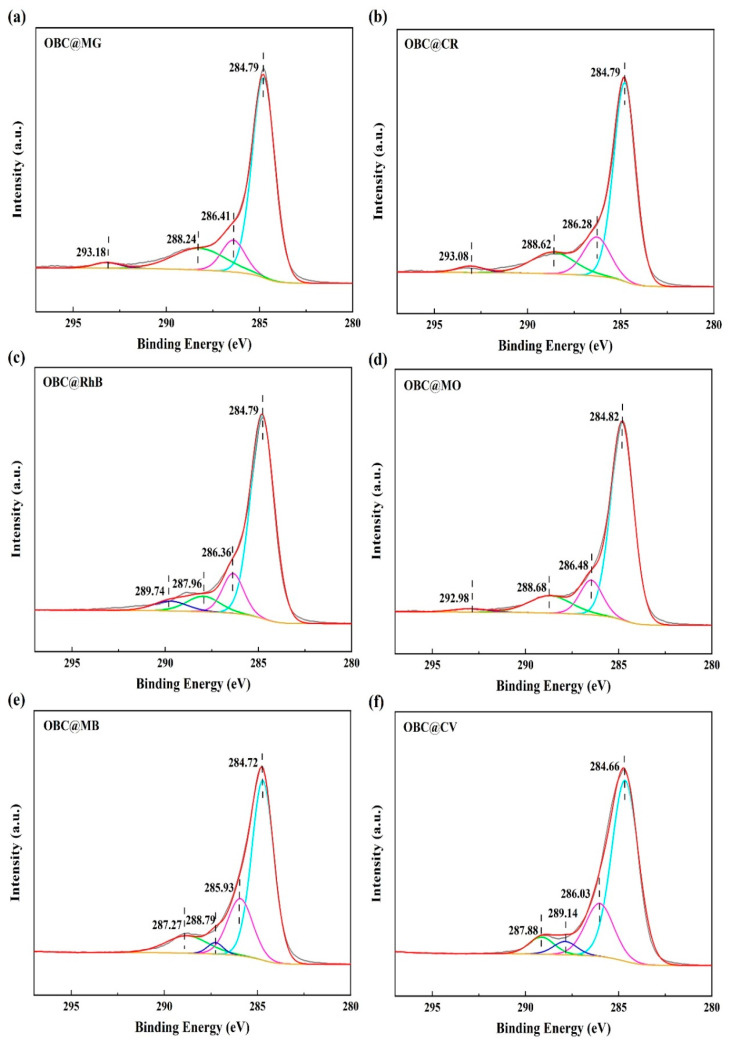
High-resolution C 1s XPS spectra: (**a**) MG, (**b**) CR, (**c**) RhB, (**d**) MO, (**e**) MB, and (**f**) CV.

**Figure 7:**
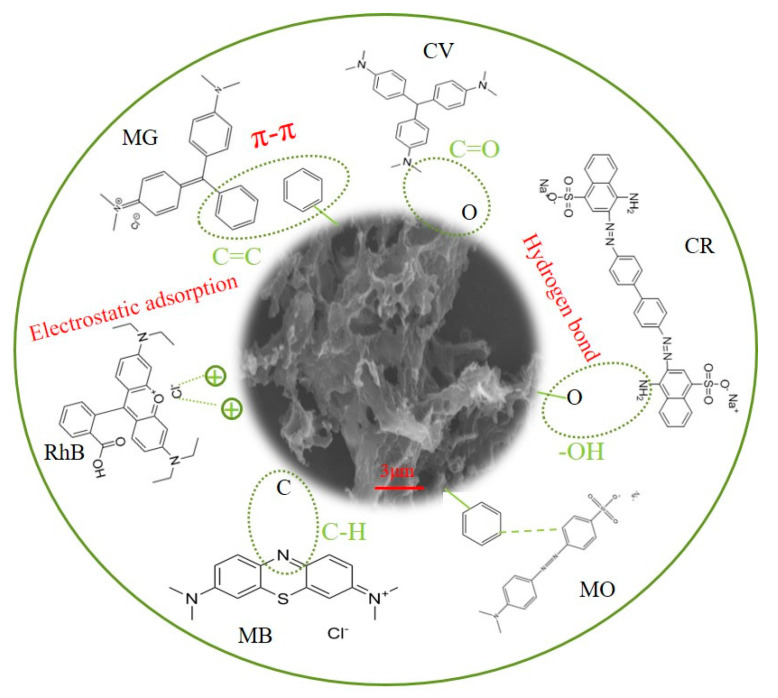
Possible mechanisms of adsorption of MG, CR, RhB, MO, MB, and CV dyes by OBC.

The high-resolution C 1s profiles of OBC after the adsorption of the six dyes MG, CR, RhB, MO, MB, and CV are shown in Figure 6. These peaks are at the cyan curve (C-C/C=C), magenta curve (C-O), green curve (O-C=O), and blue curve (π-π). The peaks of the C-O bond (286.41 eV) and the O-C=O bond (288.24 eV) were shifted in the C 1s profiles of the adsorbed MG, as shown in Figure 6a. This suggested that the -OH group was primarily responsible for the adsorption of MG. The O-C=O (288.62 and 287.96 eV) and π-π bonds (293.08 eV and 289.74 eV) of the adsorbed CR (Figure 6b) and RhB (Figure 6c) were shifted. The C 1s profiles of the adsorbed MO are shown in Figure 6d, with shifts in the C-C/C=C bonds (284.82 eV) and O-C=O bonds (288.68 eV). The aromatic ring structure of the dye molecule contributed significantly to this process by generating an accumulation of π-π bonds that took part in the reaction. The π-π (292.98 eV) and C-C/C=C bonds (284.72 eV and 284.66 eV) for the adsorbed MB (Figure 6e) and CV (Figure 6f), as well as the C-O bonds (285.93 eV and 286.03 eV), O-C=O bonds (288.79 eV and 289.14 eV), and π-π bonds (287.27 eV and 287.88 eV), were shifted, and the shifts in the peaks showed that the adsorption of the dye involved the aforementioned functional groups. Additionally, this implied that hydrogen bonding and π-π interactions were potential adsorption mechanisms and that hydroxyl groups were involved in the adsorption process. This confirmed the results of the FTIR analysis. As shown in Figure 7, we drew a diagram of the possible mechanisms of OBC’s adsorption of the MG, CR, RhB, MO, MB, and CV dyes based on the above analysis.

### 2.4. Adsorbent Regeneration

**Figure 8:**
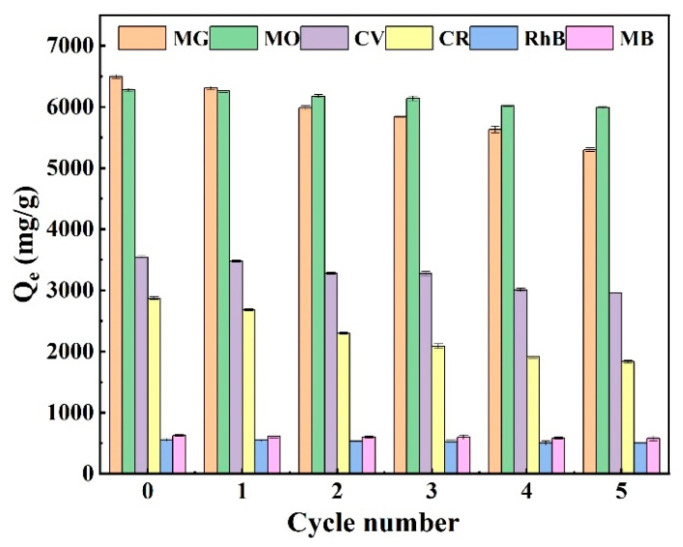
The ability of OBC to adsorb MG, CR, RhB, MO, MB, and CV at various periods in the cycle.

Because it includes both the cost of adsorption and resource recycling, the problem of adsorbent regeneration is essential for the practical treatment of wastewater. Adsorption desorption cycle tests were continuously conducted to assess the reusability of the adsorbent. To each of the six dyes (MG, CR, RhB, MO, MB, and CV, in that order), 25 mg of OBC was added. The solution’s remaining dye concentration was determined through UV spectroscopy of the supernatant, which was collected after 300 min of shaking in a shaker maintained at 303 K and 180 rpm. An anhydrous ethanol (95%, AR) solution was chosen as the desorbent. This was because, in order to facilitate desorption, the hydroxyl anion in it was able to swap with the oxygen anion in the dye molecule. The powdered material was filtered and poured into an anhydrous ethanol (95%, AR) solution, stirred for 12 h, washed repeatedly with deionized water, dried, and used for the next round of adsorption experiments. As shown in Figure 8, after five cycles, partial desorption and leftover dye molecules on the OBC’s surface or in its pore structure caused the adsorption performance to somewhat decline, but overall, there was no sharp decline, suggesting that the OBC adsorbent may be reused to save costs. A comparison of the adsorption performance of different types of adsorbent materials for dyes is shown in Table 3. Previously reported dye adsorbents were prepared under harsh conditions, through complicated processes, and at a high cost. OBC is quite effective at adsorbing MG, CR, RhB, MO, MB, and CV. It is also very easy to produce. It adsorbs a relatively wide range of dyes at a low cost and can be recycled, unlike the other related adsorbents shown in Table 3. In light of these factors the raw material sources, preparation expenses, and environmental concerns OBC is a useful adsorbent for improving dye wastewater.

## 3. Materials and Methods

### 3.1. Materials

Osmanthus fragrans was acquired at a farmers’ market. Chemicals purchased from Xilong Chemical Co. included potassium hydroxide (KOH), sodium hydroxide (NaOH, 95%), hydrochloric acid (HCl, 37%), and anhydrous ethanol (95%, AR). Methyl orange (MO, AR), methylene blue (MB, AR), rhodamine B (RhB, AR), and Congo red (CR, AR) were bought from Aladdin (Shanghai, China). Malachite green (MG, AR), and crystalline violet (CV, AR) were bought from Xilong Chemical Co., Ltd. (Shantou, China).

### 3.2. Adsorbent Preparation

Osmanthus fragrans was bought, cleaned with distilled water, dried in an oven at 80 °C, crushed into a powder, and passed through a 100-mesh sieve. In 80 mL of distilled water, 1.5 g of dried Osmanthus fragrans powder was scattered; this was transferred into a 100 mL polytetrafluorocarbon reactor and kept at 200 °C for 12 h. After that, it was washed many times with deionized water and dried in a freezer dryer. After drying, it was mixed with a KOH solution in a ratio of 1:2 by mass. The resulting slurry was dried overnight in an oven set to 80 °C. The samples were cleaned with 1 M hydrochloric acid to eliminate any last traces of inorganic pollutants. After that, they were continuously cleaned with distilled water until the solution reached pH neutrality. Finally, the samples were dried in a freeze dryer for an entire night to produce powdered OBC.

### 3.3. Characterization

The materials’ surface topography was examined by using scanning electron microscopy (SEM, HITACHI SU5000, (Tokyo, Japan)). Fourier-transform infrared spectroscopy (FTIR, Thermo Scientific iN10, (Tw, USA)) was used to examine their functional groups, and a thermal analyzer was used to evaluate their thermal stability. The phase composition of the samples was ascertained by using the X-ray powder diffraction (XRD, MiniFlex-600, (Tokyo, Japan)) technique. TG/DTA6300 (Cambridge, MA, USA) was used to measure the samples’ thermal stability. BET (Micromeritics Tristar 3000, (Atlanta, GA, USA)) was used to analyze the samples’ particular surface areas and pore structures. A UV spectrophotometer (UV-2700, (CT, Houston, TX, USA)) was used to measure the concentrations of dyes. Relevant data on the chemical state and sample surface composition were examined by using an X-ray photoelectron spectrometer (XPS, ESCAL-250XI, (Waltham, MA, USA)).

### 3.4. Adsorption Experiments

Every experiment was conducted at room temperature. The six dyes (MG, CR, MB, MO, RhB, and CV) were initially made individually, and then their reserve solutions (1000 mg/L) were diluted to different concentrations as a backup. The initial and residual concentrations of the dyes could then be determined. These sets of dye solutions, each with a different concentration, were put into quartz cuvettes and measured at 618, 498, 664, 464, 554, and 590 nm, respectively, with a UV–visible spectrophotometer to produce the calibration curves for the individual dyes. The pH of the solutions was adjusted to a range of 4–11 by using 0.1 M HCl and 0.1 M NaOH at various times (range: 5–720 min) and varied concentrations (range: 200–1000 mg·L^−1^). A total of 50 mL of the dyestuff solution (MG, CR, MB, MO, RhB, and CV) was coupled with 5 mg of OBC to evaluate the impacts of various variables on the adsorption process. Before being filtered through a 13 mm × 0.45 μm PTFE filter and having the remaining dye concentration assessed with a UV–Vis spectrophotometer, the samples were shaken at 303 K and 180 rpm on a thermostatic shaker. It was then determined how much dye was still there by measuring it with a UV–visible spectrophotometer. The data obtained at different times and initial concentrations were fitted to kinetic rate equations and adsorption isotherm models, respectively. All experiments were repeated three times to take the average, and the standard deviation was used for graphing. A series of adsorption–desorption cycle tests were conducted on the adsorbent in order to evaluate its repeatable usefulness. The adsorption capacity was calculated by using Equation (7).

(7) $$Q_{e}=\frac{C_{0}-C_{e}}{m}V$$

where *V* (L) is the volume of the dye solution, *m* (g) is the mass of the adsorbent, and *Q_e_* (mg·L^−1^) is the capacity for adsorption at the adsorption equilibrium. *C*_0_ (mg·L^−1^) and *C_e_* (mg·L^−1^) denote the dye concentration at the beginning and the dye concentration after the adsorption reached equilibrium, respectively.

## 4. Conclusions

The present study examined the starting concentration, pH, and time taken for six dyes, namely, MG, CR, RhB, MO, MB, and CV, during the process of adsorption by OBC. The batch adsorption test outcomes showed that the ideal adsorption conditions for the adsorption of various dyes were as follows: for MG, *C*_0_ = 800 mg·L^−1^, pH = 7, and *Q* = 6501.09 mg·g^−1^; for CR, *C*_0_ = 1000 mg·L^−1^, pH = 8, and *Q* = 2870.30 mg·g^−1^; for RhB, *C*_0_ = 500 mg·L^−1^, pH = 6, and *Q* = 554.93 mg·g^−1^; for MO, *C*_0_ = 1000 mg·L^−1^, pH = 7, and *Q* = 6277.72 mg·g^−1^; for MB, *C*_0_ = 700 mg·L^−1^, pH = 8, and *Q* = 626.50 mg·g^−1^; for CV, *C*_0_ = 500 mg·L^−1^, pH = 7, and *Q* = 3539.34 mg·g^−1^. The pseudo-second-order model and the Langmuir isotherm model, which more accurately depicted the full adsorption process, demonstrated that ion exchange, or chemisorption, is the primary adsorption process in monolayer adsorption. Hydrogen bonding, π-π interactions, and electrostatic adsorption are potential adsorption mechanisms. Furthermore, after five cycles, the Osmanthus fragrans biomass charcoal’s adsorption ability did not abruptly decline, indicating that it may have application as a possible adsorbent for dye adsorption.

## Figures and Tables

**Table 1:** The OBC’s adsorption of the MG, CR, RhB, MO, MB, and CV dyes for the fitting of pseudo-first-order and pseudo-second-order kinetic models with specific parameters.

Adsorbent	Pseudo-First-Order Model			Pseudo-Second-Order Model		
	*K*_1_ (min^−1^)	*Q_e_* (mg·g^−1^)	*R* ^2^	*K*_2_ (g·mg^−1^·min^−1^)	*Q_e_* (mg·g^−1^)	*R* ^2^
MG	2.174 × 10^−2^	4062.620	0.899	7.398 × 10^−6^	4443.008	0.994
CR	1.037 × 10^−2^	1696.880	0.958	6.410 × 10^−6^	1966.689	0.997
RhB	10.176 × 10^−2^	477.290	0.839	290.60 × 10^−6^	508.968	0.998
MO	2.100 × 10^−2^	2661.941	0.975	9.470 × 10^−6^	2969.427	0.999
MB	9.051 × 10^−2^	454.027	0.752	246.071 × 10^−6^	490.371	0.997
CV	7.939 × 10^−2^	2292.463	0.839	44.942 × 10^−6^	2468.242	0.998

**Table 2:** Adsorption isotherm parameters for the adsorption of MG, CR, RhB, MO, MB, and CV onto the OBC.

Models	Parameters	MG	CR	RhB	MO	MB	CV
Langmuir	*Q_m_* (mg·g^−1^)	7386.845	3209.326	608.709	6577.761	692.476	3069.929
	*K_L_* (L·mg^−1^)	456.844	338.953	90.949	837.915	137.184	117.323
	*R* ^2^	0.996	0.998	0.997	0.987	0.996	0.997
Freundlich	1/*n*	0.591	0.512	0.154	0.910	0.220	0.194
	*K_F_* (mg·g^−1^)·(L·mg^−1^)^1/*n*^	106.688	85.851	194.074	10.347	135.461	732.094
	*R* ^2^	0.956	0.962	0.957	0.975	0.964	0.961
Temkin	*K_T_* (L·g^−1^)	0.011	0.014	0.988	0.006	0.163	0.289
	*B* (J·mol^−1^)	2616.080	1136.487	81.854	2805.106	121.485	492.192
	*R* ^2^	0.981	0.985	0.963	0.976	0.970	0.966

**Table 3:** Comparison of the adsorption capacity of OBC for MG, CR, RhB, MO, MB, and CV dyes with that of other adsorbents.

Adsorbent	*Q_m_* (mg·g^−1^)						Ref.
	MG	CR	MB	MO	RhB	CV	
DBSA-Fe_3_O_4_@BC					367.67		[33]
MNB		1360.00			320.00		[20]
HLP	2468.00	404.40					[24]
SWAC			249.00				[25]
HTCM			124.22	1009.66	136.35	124.87	[43]
MIL-121		597.90	246.00				[44]
TpStb-SO_3_H	5857.00		1078.00			1861.00	[45]
MnO_2_-NP-CPC	319.40		289.84		267.67		[46]
CGLC		220.70			572.80		[47]
Alg-g-PANI	578.30	409.60					[48]
PVP/rGO/CFO		355.90	333.30		558.70		[49]
OBC	6501.09	2870.30	626.50	6277.72	554.93	3539.34	This work

## Data Availability

The data can be made available upon reasonable request.
